# Treponemal Antibody Seroprevalence Using a Multiplex Bead Assay from Samples Collected during the 2018 Nigeria HIV/AIDS Indicator and Impact Survey: Searching for Yaws in Nigeria

**DOI:** 10.4269/ajtmh.22-0670

**Published:** 2023-04-10

**Authors:** Sarah Anne J. Guagliardo, Nishanth Parameswaran, Ndidi Agala, Ado Abubakar, Gretchen Cooley, Tun Ye, Mary Kamb, Nwando Mba, Nwachukwu William, Stacie Greby, Nnaemeka Iriemenam, Matthias Alagi, McPaul Okoye, Diana Martin

**Affiliations:** 1 Division of Parasitic Diseases and Malaria, US Centers for Disease Control and Prevention, Atlanta, Georgia;; 2 Institute of Human Virology, Abuja, Nigeria;; 3 Center for Global Health, US Centers for Disease Control and Prevention, Atlanta, Georgia;; 4 National Reference Laboratory, Nigeria Centre for Disease Control, Abuja, Nigeria;; 5 Division of Global HIV and TB, US Centers for Disease Control and Prevention, Abuja, Nigeria

## Abstract

Yaws is a chronic, relapsing disease of skin, bone, and cartilage caused by *Treponema pallidum* subsp. *pertenue*. Yaws was last reported in Nigeria in 1996, although neighboring countries have recently reported cases. We investigated serological evidence for yaws among children aged 0–14 years in Nigeria by measuring antibodies to the treponemal antigens rp17 and TmpA in blood specimens from a 2018 nationally representative HIV survey using a multiplex bead assay. The presence of antibodies to both antigens (“double positive”) likely reflects current or recent treponemal infection. Overall, 1.9% (610/31,549) of children had anti-TmpA antibodies, 1.5% (476/31,549) had anti-rp17 antibodies, and 0.1% (39/31,549) were double positive. Among households, 0.5% (84/18,021) had a double-positive child, with a clustering of double-positive children. Although numbers are low, identification of antibodies to both TmpA and rp17 may warrant investigation, including more granular epidemiologic and clinical data, to assess the potential for continuing yaws transmission in Nigerian children.

Yaws is a chronic, relapsing, nonvenereal treponematosis caused by the bacterium *Treponema pallidum* subsp. *pertenue*.^1^ Yaws is transmitted via direct skin contact, affects the skin, bone, and cartilage,^1^ and is typically found in warm, humid, and tropical areas.^1^ Seventy-five percent of cases occur in children < 15 years of age, with peak incidence in 6 to 10 year olds.^1^ During the 1950 s and 1960 s, yaws was the subject of eradication campaigns using injectable benzathine penicillin in 46 endemic countries.^2^ These campaigns treated over 50 million yaws cases and led to a 95% decrease in global yaws burden,^2^ but eradication was not achieved. Yaws eradication efforts were reinvigorated after a 2012 study that showed that a single oral dose of azithromycin was as effective as injectable penicillin at treating yaws.^3^ Achievement of the WHO 2030 eradication goal for yaws will require identification of all areas of ongoing transmission.^4^ Currently, global surveillance reporting is uneven across countries, and 84 countries fall into the category of “previously endemic, current status unknown”^5^; one of these countries is Nigeria.

In Nigeria, the standard case definition of yaws requires a history of residence in an endemic area (past or present) and presentation of clinically active (visible) yaws lesions. A confirmed case is “a suspected case with a positive serological test (rapid treponemal test for syphilis confirmed by a dual-path platform test)” (242_1601639437.pdf; https://ncdc.gov.ng). Yaws was thought to be eliminated from Nigeria in the 1950s^6^ but several cases have since been identified, including 64 cases of yaws—based on suggestive clinical signs confirmed with serologic testing—during a filariasis survey in Garkida, northeast Nigeria in 1999, representing 4.2% of the population surveyed.^7^ Yaws has also been reported in neighboring countries such as Ghana, Cote d’Ivoire, Benin, and Cameroon as recently as 2021.^8^^,^^9^

To obtain serological data on recent yaws transmission in Nigeria, we measured antibodies against two treponemal antigens in dried blood spot (DBS) specimens collected during the 2018 Nigeria HIV/AIDS Indicator and Impact Survey (NAIIS). Nigeria HIV/AIDS Indicator and Impact Survey was a population-based survey designed to estimate HIV prevalence and viral load suppression at the national and subnational levels and HIV incidence at the subnational level.^10^ Nigeria HIV/AIDS Indicator and Impact Survey used a two-stage stratified cluster sample design, selecting enumeration areas (EAs) followed by households. Data collection occurred between July and December 2018, enrolling 225,169 participants from 97,250 randomly selected households in 4,035 nationally representative sample clusters across 36 states and the Federal Capital Territory. Children were included in the study in approximately 25% of households to provide a representative national estimate of pediatric HIV prevalence. DBS specimens from individuals with parental consent (and assent for older children) were stored in −80 °C freezers for future testing. Stored DBSs were tested for antibodies against 37 antigens from 17 pathogens using a multiplex bead assay as part of the Nigeria Multi-Disease Serologic Surveillance Using Stored Specimens (NMS4) study. The panel included two treponemal antigens: rp17, representing historical infection (similar to *T. pallidum* particle agglutination), and TmpA, indicative of current or recent infection (similar to the rapid plasma reagin test). This study was reviewed and approved by human subject reviewers of the National Health Research Ethics Committee of Nigeria, the University of Maryland Baltimore, and the US Centers for Disease Control and Prevention.

Cutoffs for positivity for each antigen were determined using receiver operating characteristic (ROC) curves comparing responses from 84 DBSs from a yaws-endemic setting in Vanuatu and 74 serum samples from a nonendemic setting in the United States serving as controls. A high-titer serum was then used to generate a dilution curve of arbitrary units versus the median fluorescence intensity (MFI) run at the National Reference Laboratory in Abuja.

Because these antibody tests do not differentiate yaws from syphilis, analysis was restricted to 1 to 14 year olds (*N* = 31,549). Also included was a sample of 9,750 women of reproductive age (WRAs; aged 15–44 years, included in NMS4 to estimate tetanus vaccination coverage in that population) to link with children for possible evidence of maternal transmission. After data cleaning, we tabulated the number of 1 to 14 year olds who had positive antibody responses to TmpA, rp17, and both antigens (“double positive”). The age and sex of children who were double positive were compared with those who were seronegative for both antigens by χ^2^ tests. The MFI with background subtracted out (MFI-bg) of double-positive children was compared with single-positive children using a Wilcoxon rank-sum test. The number of double-positive children in the same household with a seropositive WRA by any antigen was determined, as was the number of households with multiple occurrences of a seropositive child by any antigen. Finally, geographic clustering was assessed by tallying the number of double-positive children or WRAs that occurred within the same EA.

Among 1 to 14 year olds, 39 (0.12%) were double positive, 570 (1.8%) had antibodies to only TmpA, and 437 (1.4%) had antibodies to only rp17 (Table 1). For both TmpA and rp17, 5 to 10 year olds were more likely to be seropositive than 0 to 4 year olds or 11 to 15 year olds (χ^2^ = 84.26, *P* < 0.0001). No differences by age were observed for double-positive children compared with seronegative children. MFI-bg among double-positive children was not higher than single-positive children for rp17 (*P* > 0.999) or TmpA (*P* = 0.999) (Figure 1). Based on the ROC curves, the specificity for each antigen was 99%, which means these may represent false-positive results. We therefore looked for clustering of double-positive samples, because a high likelihood of recent treponemal infection requires positivity on both tests.

**Table 1:** Demographic characteristics of seropositive children < 15 years old

Characteristic	TmpA (*N* = 610; 1.93%)				rp17 (*N* = 476; 1.51%)				Double positive (*N* = 39; 0.12%)			
	*n*	%	χ^2^	*P*	*n*	%	χ^2^	*P*	*n*	%	χ^2^	*P*
Sex												
Female	331	45.74	2.57	> 0.10	227	47.70	0.31	> 0.58	14	35.90	2.22	0.14
Male	279	54.26			249	52.31			25	64.10		
Age (years)												
0–4	82	13.44	**84.26**	**< 0.0001**	180	37.82	**14.78**	**< 0.001**	7	17.95	3.42	0.18
5–10	348	57.05			192	40.34			20	51.28		
11–14	180	29.51			104	21.85			12	30.77		

No statistically significant differences were found by sex. We compared children who were seropositive for both antibody responses with children who were negative for both antibodies. Bolding indicates statistical significance.

**Figure 1.**
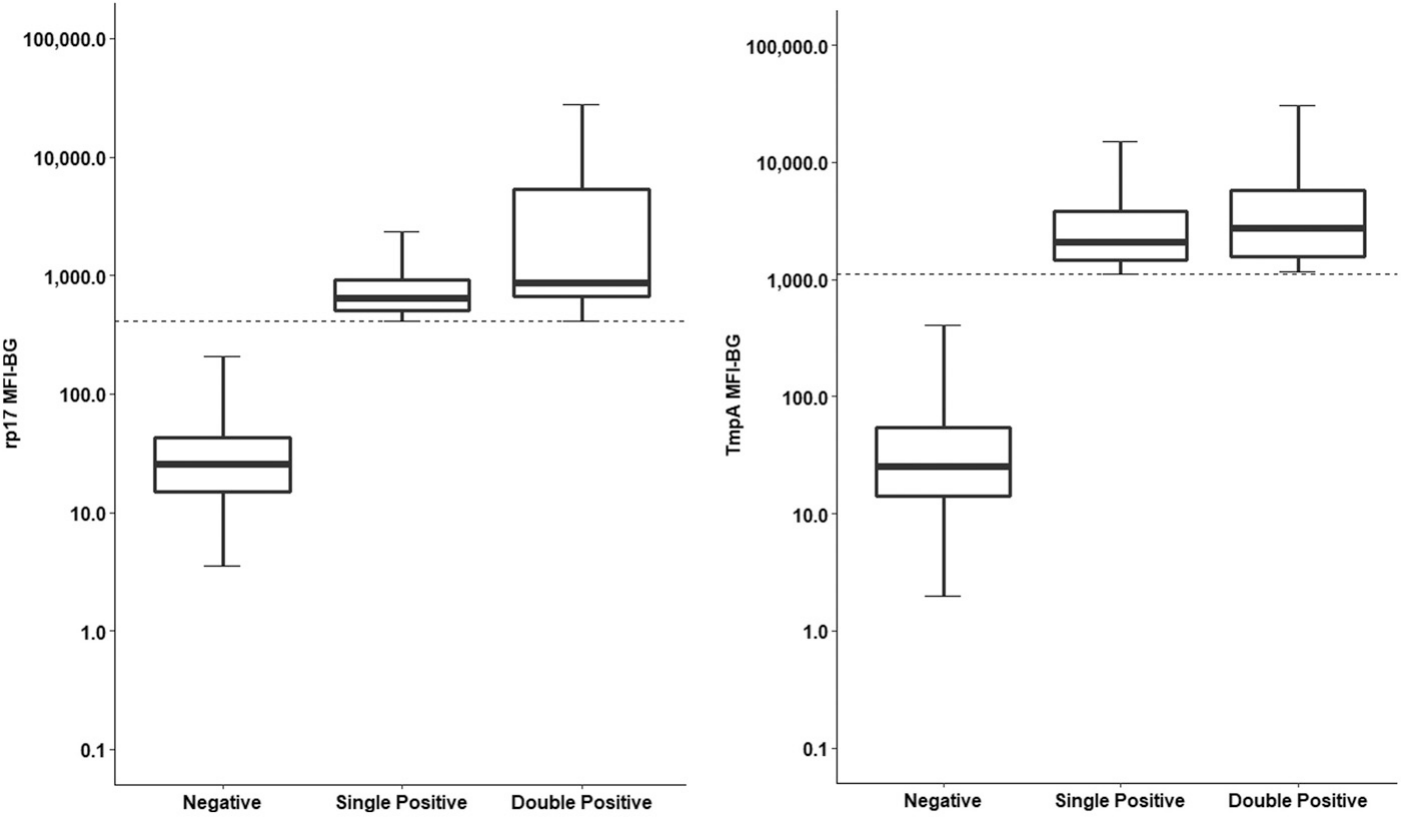
Antibody levels against treponemal antigens rp17 and TmpA. The graphs show the median fluorescence intensity (MFI) with background subtracted (MFI-bg) of antibodies against rp17 (left) and TmpA (right) for samples testing negative for rp17 (left; *N* = 30,983) or TmpA (right; *N* = 30,849), positive for only rp17 (*N* = 437) or TmpA (*N* = 570), or positive to both antigens (*N* = 39). The *y* axis is shown on a log_10_ scale.

No household had more than one double-positive child, and no double-positive child lived in the same household with a WRA that had antitreponemal antibodies. Eighty-one of 3,447 EAs had double-positive children (*N* = 38 EAs) or WRAs (*N* = 43 EAs). Three EAs had two double-positive children or WRAs: one EA had double-positive children aged 5 and 14 years (Figure 2) in different households and the other two EAs had a double-positive WRA.

**Figure 2.**
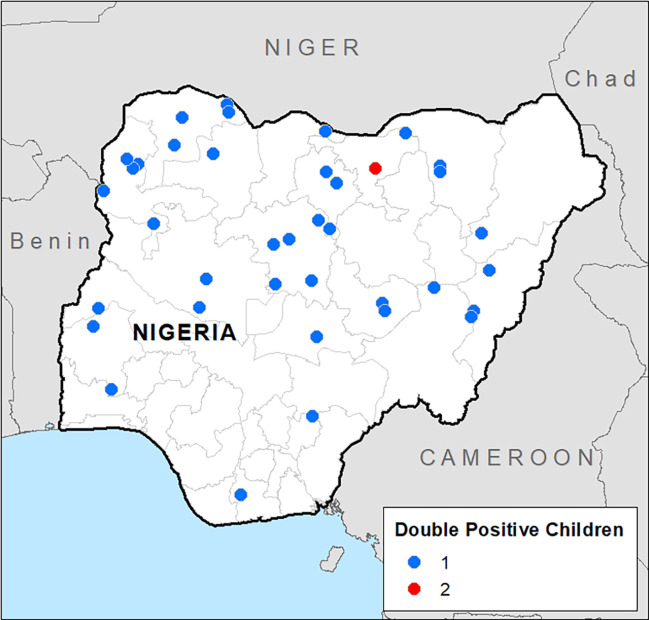
Map of children seropositive for antibodies to rp17, TmpA, or both. Circles indicate the location of children with a double-positive test for rp17 and TmpA. The color of the circle indicates the number of children in each cluster with a double-positive test as indicated in the legend.

The low proportion of 1 to 14 year olds with positive serology against both antigens and the lack of significant clustering of double-positive tests suggests very little yaws among the sampled population. Because of the low positive predictive value of testing in low-prevalence settings, and because positive tests may reflect other treponemal infections (e.g., syphilis), case definitions for yaws involve detailed clinical assessments.^11^ In this study, in the absence of clinical data or more detailed epidemiologic data, the results do not definitively exclude the possibility of ongoing yaws transmission in Nigeria. Here, we discuss demographic, serologic, and geographic patterns in our findings and consider whether they suggest yaws transmission.

Although 5 to 10 year olds—the age range in which peak incidence of yaws should occur—were more likely to be positive for antibodies to either TmpA or rp17 than children in other age ranges in this study, this pattern was not observed in children with a double-positive test. Males may have increased risk of yaws,^12^ but we saw no differences in double seropositivity by sex. The low numbers of double-positive individuals detected indicate at least some findings might represent false-positive results. Double-positive specimens did not have higher MFI-bg than single-positive tests, which might be expected if these specimens were true positives.

The WHO has set a 2030 target for yaws eradication, and current WHO guidance recommends that serosurveys be conducted in 1 to 5 year olds in yaws- and non-yaws-endemic villages for 3 consecutive years, alongside active case searches.^11^ Despite this goal, very little funding support is currently available for identifying areas of active yaws transmission. The ability to leverage nonyaws surveys would greatly benefit yaws programs to identify areas of ongoing yaws transmission in need of surveillance and intervention, particularly in countries, such as Nigeria, in which current endemicity is not known. Our results highlight the potential utility—as well as some concerns—of using multiplex serology for integrated testing on the basis of available sera from nonyaws surveys for this purpose. The antigens rp17 and TmpA are not currently in WHO guidance for yaws programs, but have shown performance comparable to traditional treponemal and nontreponemal serology.^13^^,^^14^ However, surveys should be designed to identify foci of transmission; even the massive NAIIS national sample lacked the spatial scale to identify foci of yaws transmission. Additionally, yaws transmission is considered interrupted when serosurveys provide evidence of continuous negative rapid treponemal tests for at least 3 consecutive years in samples of asymptomatic 1 to 5 year olds in the community.^11^ Therefore, any single cross-sectional survey (regardless of size) is inadequate to confirm interruption of transmission. The reliance on sampling not designed to measure the prevalence of this highly focal neglected tropical disease, coupled with the slow adoption of new testing and targets by WHO programs, is an important limitation. Ideally, data from a cross-sectional serosurvey could be followed by targeted active case finding in areas where children with positive serology reside.

## References

[1] World Health Organization , 2021. *Yaws (Endemic Trepanematoses)*. Available at: https://www.who.int/health-topics/yaws#tab=tab_1. Accessed March 20, 2023.

[2] Asiedu K Fitzpatrick C Jannin J, 2014. Eradication of yaws: historical efforts and achieving WHO’s 2020 target. PLoS Negl Trop Dis 8: e3016. 25254372 10.1371/journal.pntd.0003016 PMC4177727

[3] Mitja O Hays R Ipai A Penias M Paru R Fagaho D de Lazzari E Bassat Q, 2012. Single-dose azithromycin versus benzathine benzylpenicillin for treatment of yaws in children in Papua New Guinea: an open-label, non-inferiority, randomised trial. Lancet 379: 342–347. 22240407 10.1016/S0140-6736(11)61624-3

[4] World Health Organization , 2012. Eradication of yaws—the Morges strategy. Wkly Epidemiol Rec 87: 189–194. 24340400

[5] Mitja O Asiedu K Mabey D, 2013. Yaws. Lancet 381: 763–773. 23415015 10.1016/S0140-6736(12)62130-8

[6] Zahra A, 1956. Yaws eradication campaign in Nsukka division, eastern Nigeria. Bull World Health Organ 15: 911–935. 13404467 PMC2538174

[7] Akogun OB, 1999. Yaws and syphilis in the Garkida area of Nigeria. Zentralbl Bakteriol 289: 101–107. 10096171 10.1016/s0934-8840(99)80130-3

[8] Ndzomo Ngono JP Tchatchouang S Noah Tsanga MV Njih Tabah E Tchualeu A Asiedu K Giacani L Eyangoh S Crucitti T, 2021. Ulcerative skin lesions among children in Cameroon: it is not always yaws. PLoS Negl Trop Dis 15: e0009180. 33591973 10.1371/journal.pntd.0009180 PMC7909670

[9] Okine RNA Sarfo B Adanu RM Kwakye-Maclean C Osei FA, 2020. Factors associated with cutaneous ulcers among children in two yaws-endemic districts in Ghana. Infect Dis Poverty 9: 26. 32160927 10.1186/s40249-020-00641-2 PMC7066816

[10] National Agency for the Control of HIV/AIDS, 2018. *Nigeria HIV/AIDS Indicator and Impact Survey*. Available at: https://www.naiis.ng/. Accessed May 13, 2021.

[11] World Health Organization , 2018. *Eradication of Yaws: A Guide for Programme Managers*. Available at: https://apps.who.int/iris/bitstream/handle/10665/259902/9789241512695-eng.pdf?sequence=1&isAllowed=y. Accessed March 20, 2023.

[12] Kazadi WM Asiedu KB Agana N Mitja O, 2014. Epidemiology of yaws: an update. Clin Epidemiol 6: 119–128. 24729728 10.2147/CLEP.S44553 PMC3979691

[13] Cooley GM , 2016. Evaluation of multiplex-based antibody testing for use in large-scale surveillance for yaws: a comparative study. J Clin Microbiol 54: 1321–1325. 26962086 10.1128/JCM.02572-15 PMC4844712

[14] Parameswaran N , 2021. Antibody responses to two recombinant treponemal antigens (rp17 and TmpA) before and after azithromycin treatment for yaws in Ghana and Papua New Guinea. J Clin Microbiol 59: e02509-20. 33568467 10.1128/JCM.02509-20 PMC8091825

